# Dunn Index Bootstrap (DIBS): A procedure to empirically select a cluster analysis method that identifies biologically and clinically relevant molecular disease subgroups

**DOI:** 10.1186/1471-2105-16-S15-P12

**Published:** 2015-10-23

**Authors:** Iwona Pawlikowska, Zhifa Liu, Lei Shi, Tong Lin, Tanja Gruber, Giles Robinson, Arzu Onar-Thomas, Stan Pounds

**Affiliations:** 1 Department of Biostatistics, St. Jude Children's Research Hospital, Memphis, TN 38105, USA; 2 Department of Oncology, St. Jude Children's Research Hospital, Memphis, TN 38105, USA; 3 Institue of Mathematics, University of Silesia, Katowice, 2469011, Poland

## Background

Cluster analysis is widely used in cancer research to discover molecular subgroups that inform subsequent laboratory investigations and define risk classification criteria for subsequent clinical trials. However, for any data set, there are a very large number of candidate cluster analysis methods (CCAMs) due to the many choices for feature selection criteria, number of selected features, number of clusters to define, etc. Frequently, a specific CCAM is chosen without quantifying the validity of its results in terms of reproducibility or distinctiveness of the reported subgroups.

## Materials and methods

Here, we propose the Dunn Index Bootstrap (DIBS) procedure to quantify the reproducibility and distinctiveness of subgroups defined by many CCAMs. DIBS applies each CCAM to the observed data and many bootstrap data sets obtained by subject resampling. The bootstrap results are used to compute metrics of subgroup reproducibility and distinctiveness of the subgroups defined by each CCAM.

## Results

DIBS was used to characterize the performance of each of 4,032 CCAMs in the analysis of one RNA-seq, two microarray gene expression, and one methylation array data set from three different cancers. In each example, DIBS identified specific CCAMs that defined subgroups of well-established biological and clinical relevance.

